# Impact of Blood Pressure Visit‐to‐Visit Variability on Adverse Events in Patients With Nonvalvular Atrial Fibrillation: Subanalysis of the J‐RHYTHM Registry

**DOI:** 10.1161/JAHA.120.018585

**Published:** 2020-12-29

**Authors:** Eitaro Kodani, Hiroshi Inoue, Hirotsugu Atarashi, Ken Okumura, Takeshi Yamashita, Toshiaki Otsuka, Hideki Origasa, the J‐RHYTHM Registry Investigators, H Inoue, K Okumura, H Atarashi, T Yamashita, M Sakurai, Y Kawamura, K Okumura, I Kubota, Y Kaneko, K Matsumoto, S Ogawa, H Atarashi, T Yamashita, H Inoue, Y Aizawa, I Kodama, E Watanabe, Y Koretsune, Y Okuyama, A Shimizu, O Igawa, S Bando, M Fukatani, T Saikawa, A Chishaki, H Origasa, N Kato, K. Kanda, J Kato, H Obata, M Aoki, H. Honda, Y Konta, T Hatayama, Y Abe, K Terata, T Yagi, A Ishida, T Komatsu, H Tachibana, H Suzuki, Y Kamiyama, T Watanabe, M Oguma, M Itoh, O Hirono, Y Tsunoda, K Ikeda, T Kanaya, K Sakurai, H Sukekawa, S Nakada, T Itoh, S Tange, M. Manita, M Ohta, H Eguma, R Kato, Y Endo, T Ogino, M Yamazaki, H Kanki, M Uchida, S Miyanaga, K Shibayama, N Toratani, T Kojima, M Ichikawa, M Saito, Y Umeda, T Sawanobori, H Sohara, S Okubo, T Okubo, T. Tokunaga, O Kuboyama, H Ito, Y Kitahara, K Sagara, T Satoh, E Kodani, K Sugi, Y Kobayashi, Y Higashi, T Katoh, Y Hirayama, N Matsumoto, M Takano, T Ikeda, S Yusu, S Niwano, Y Nakazato, Y Kawano, M Sumiyoshi, N Hagiwara, K Murasaki, H Mitamura, S Nakagawa, K Okishige, K Azegami, H Aoyagi, K Sugiyama, M Nishizaki, N Yamawake, I Watanabe, K Ohkubo, H Sakurada, S Fukamizu, M Suzuki, W Nagahori, T Nakamura, Y Murakawa, N Hayami, K Yoshioka, M Amino, K Hirao, A Yagishita, K Ajiki, K Fujiu, Y Imai, A Yamashina, T Ishiyama, M Sakabe, K Nishida, H Asanoi, H Ueno, Lee, Y Mitsuke, H Furushima, K Ebe, M Tagawa, M Sato, M. Morikawa, K Yamashiro, K Takami, T Ozawa, M Watarai, M Yamauchi, H Kamiya, H. Hirayama, Y. Yoshida, T Murohara, Y Inden, H Osanai, N Ohte, T Goto, I Morishima, T Yamamoto, E Fujii, M Senga, H. Hayashi, T Urushida, Y Takada, R Kato, N Tsuboi, T Noda, T Hirose, T Onodera, S Kageyama, T Osaka, T. Tomita, K Shimada, M Nomura, H Izawa, A Sugiura, T Arakawa, K. Kimura, T Mine, T Makita, H Mizuno, A Kobori, T Haruna, M Takagi, T Watanabe, N Tanaka, H Shimizu, T Kurita, K Motoki, N Takeda, Y Kijima, M Ito, A Nakata, Y Ueda, A Hirata, S Kamakura, K Satomi, T Noda, Y. Yamada, Y. Yoshiga, H Ogawa, M Kimura, T Hayano, T Kinbara, H Tatsuno, M Harada, K. Kusano, M Adachi, A Yano, M Sawaguchi, J Yamasaki, T Matsuura, Y Tanaka, H Moritani, T Maki, S Okada, M Takechi, T Hamada, A Nishikado, Y Takagi, I Matsumoto, T Yamamoto, T Soeki, Y Doi, M Okawa, H Seo, S Kitamura, K Yamamoto, M Akizawa, N Kaname, S Ando, S Narita, T Nakamura, T Inou, Y Fukuizumi, K Saku, M Ogawa, Y Urabe, M Ikeuchi, S Harada, H Yamabe, Y Imamura, Y. Yamanouchi, K Sadamatsu, K Yoshida, T Kubota, N Takahashi, N Makino, Y Higuchi, T Ooie, T Iwao, K. Kitamura, T Imamura, K Maemura, N Komiya, M Hayano, H Yoshida, K Yamashiro, K. Kumagai

**Affiliations:** ^1^ Department of Internal Medicine and Cardiology Nippon Medical School Tama‐Nagayama Hospital Tokyo Japan; ^2^ Saiseikai Toyama Hospital Toyama Japan; ^3^ Minami‐Hachioji Hospital Tokyo Japan; ^4^ Saiseikai Kumamoto Hospital Kumamoto Japan; ^5^ The Cardiovascular Institute Tokyo Japan; ^6^ Department of Hygiene and Public Health Nippon Medical School Tokyo Japan; ^7^ Division of Biostatistics and Clinical Epidemiology University of Toyama Japan

**Keywords:** atrial fibrillation, blood pressure, major hemorrhage, thromboembolism, variability, Atrial Fibrillation, Hypertension

## Abstract

**Background:**

Blood pressure (BP) variability has reportedly been a risk factor for various clinical events. To clarify the influence of BP visit‐to‐visit variability on adverse events in patients with nonvalvular atrial fibrillation, a post hoc analysis of the J‐RHYTHM Registry was performed.

**Methods and Results:**

Of 7406 outpatients with nonvalvular atrial fibrillation from 158 institutions, 7226 (age, 69.7±9.9 years; men, 70.7%), in whom BP was measured 4 times or more (14.6±5.0 times) during the 2‐year follow‐up period or until occurrence of an event, constituted the study group. SD and coefficient of variation of BP values were calculated as BP variability. Thromboembolism, major hemorrhage, and all‐cause death occurred in 110 (1.5%), 121 (1.7%), and 168 (2.3%) patients, respectively. When patients were divided into quartiles of systolic BP‐SD (<8.20, 8.20–10.49, 10.50–13.19, and ≥13.20 mm Hg), hazard ratios (HRs) for all adverse events were significantly high in the highest quartile compared with the lowest quartile (HR, 2.00, 95% CI, 1.15–3.49, *P*=0.015 for thromboembolism; HR, 2.60, 95% CI, 1.36–4.97, *P*=0.004 for major hemorrhage; and HR, 1.85, 95% CI, 1.11–3.07, *P*=0.018 for all‐cause death) after adjusting for components of the CHA_2_DS_2_‐VASc score, warfarin and antiplatelet use, atrial fibrillation type, BP measurement times, and others. These findings were consistent when BP‐coefficient of variation was used instead of BP‐SD.

**Conclusions:**

Systolic BP visit‐to‐visit variability was significantly associated with all adverse events in patients with nonvalvular atrial fibrillation. Further studies are needed to clarify the causality between BP variability and adverse outcomes in patients with nonvalvular atrial fibrillation.

**Registration:**

URL: https://www.umin.ac.jp/ctr/; Unique Identifier: UMIN000001569.

**Nonstandard Abbreviations and Acronyms**
AFFIRM: Atrial Fibrillation Follow‐Up Investigation of Rhythm Management
BP‐end: blood pressure at the time closest to an event or at the end of follow‐up
CrCl: creatinine clearance
CV: coefficient of variation
NVAF: nonvalvular atrial fibrillation
PT‐INR: prothrombin time international normalized ratio
TTR: time in therapeutic range

**Clinical Perspective**

**What Is New?**

- Systolic blood pressure (BP) visit‐to‐visit variability was significantly associated with the increased risk of thromboembolism, major hemorrhage, and all‐cause death in patients with nonvalvular atrial fibrillation, independent of BP values at the closest time to an event and other conventional risk factors.
- Systolic BP visit‐to‐visit variability was superior to systolic BP values at the closest time to an event as a predictor of major hemorrhage and composite events.
- In Japanese patients receiving warfarin, BP variability was significantly correlated with time in therapeutic range in those aged ≥70 years and prothrombin time international normalized ratio variability.

**What Are the Clinical Implications?**

- Systolic BP visit‐to‐visit variability is evidently important for the prevention of all adverse events in patients with nonvalvular atrial fibrillation as well as in patients without atrial fibrillation.
- BP visit‐to‐visit variability as well as anticoagulation quality (time in therapeutic range) and intensity (prothrombin time international normalized ratio) should be taken into account for the management of patients with nonvalvular atrial fibrillation.

Hypertension is an established risk factor for several cardiovascular diseases^1^, ^2^, ^3^ including atrial fibrillation (AF).^4^, ^5^ In patients with AF, hypertension is one of the risk factors for thromboembolism and hemorrhagic complications.^6^, ^7^ Thus, it has been adopted as a component of widely used traditional risk scores.^8^, ^9^, ^10^ However, hypertension is not always detected as an independent risk factor for thromboembolism or hemorrhagic complications,^11^, ^12^, ^13^, ^14^, ^15^ probably because of leaving blood pressure (BP) control status and variability during the follow‐up period out of consideration. Indeed, our previous report in patients with nonvalvular AF (NVAF) demonstrated that either clinical diagnosis of hypertension (prior history, use of antihypertensive drugs, or both) or baseline BP values did not emerge as an independent predictor of thromboembolism. However, systolic BP values (≥136 mm Hg) at the time closest to an event or at the end of follow‐up (BP‐end) were significantly associated with the incidence of both thromboembolism and major hemorrhage.^7^

BP visit‐to‐visit variability, as an index of long‐term BP variability,^16^ has reportedly been a risk factor for various clinical events and mortality in patients with hypertension as well as in the general population.^17^, ^18^, ^19^, ^20^, ^21^ However, the influence of BP variability on adverse events in patients with NVAF has not been sufficiently elucidated.^22^ Therefore, in order to clarify the influence of BP visit‐to‐visit variability on adverse events such as thromboembolism, major hemorrhage, and all‐cause death, a post hoc analysis was performed using the SD and coefficient of variation (CV) of BP values during the follow‐up period in the J‐RHYTHM Registry.

## Methods

The data that support the findings of this study are available from the corresponding author upon reasonable request from an appropriately qualified research group.

### Study Design of the J‐RHYTHM Registry

The J‐RHYTHM Registry was conducted as a nationwide prospective observational study to investigate the status of anticoagulation therapy and the optimal anticoagulation therapy in Japanese patients with AF.^23^ The study design and baseline patient characteristics have been reported elsewhere.^23^, ^24^ Briefly, the study protocol conformed to the Declaration of Helsinki and was approved by the ethics committee of each participating institution. Written informed consent was obtained from all participants at the time of enrollment. A consecutive series of outpatients with AF of any type was enrolled from 158 institutions, regardless of the use of antihypertensive drugs. All drugs and their dosages were selected at the discretion of the treating physicians. Patients with valvular AF (mechanical heart valve and mitral stenosis)^25^ were excluded from this subanalysis. Seated brachial BP was measured in each patient at the time of enrollment (baseline) and at each follow‐up visit by either the auscultatory method or an automated sphygmomanometer, as appropriate at each institution. For the present post hoc analysis, patients with AF, in whom BP was measured 4 times or more during the 2‐year follow‐up period or until occurrence of an event, were included.

Anticoagulation intensity was determined at the time of enrollment (baseline) and at each follow‐up visit using the prothrombin time international normalized ratio (PT‐INR) in patients receiving warfarin. Time in therapeutic range (TTR) was calculated by the Rosendaal method^26^ to evaluate the overall quality of anticoagulation therapy during the follow‐up period. In this study, the target PT‐INR level was set at 1.6 to 2.6 for elderly patients aged ≥70 years and at 2.0 to 3.0 for patients aged <70 years according to the Japanese guidelines.^27^

### Follow‐Up and Definition of End Points

Patients were followed up for 2 years or until an event, whichever occurred first. The primary end points were as follows: thromboembolism including symptomatic ischemic stroke, transient ischemic attack, and systemic embolic events; major hemorrhage including intracranial hemorrhage, gastrointestinal hemorrhage, and other hemorrhages requiring hospitalization; and all‐cause death. The composite of thromboembolism, major hemorrhage, and all‐cause death, whichever occurred first for each patient, was also evaluated. The diagnostic criteria for each event have been described elsewhere.^23^, ^24^

### Evaluation of BP Variability and Grouping of Patients

According to a post hoc analysis of the AFFIRM (Atrial Fibrillation Follow‐Up Investigation of Rhythm Management) Study,^22^ SD and CV (=SD/mean) of BP values were calculated as an index of BP visit‐to‐visit variability. Patients were categorized in quartiles of systolic BP‐SD (<8.20, 8.20–10.49, 10.50–13.19, and ≥13.20 mm Hg) or diastolic BP‐SD (<5.80, 5.80–7.29, 7.30–9.29, and ≥9.30 mm Hg). Using BP‐CV instead of BP‐SD, patients were also categorized in quartiles of systolic BP‐CV (<6.6, 6.6–8.3, 8.4–10.4, and ≥10.5%) or diastolic BP‐CV (<7.9, 7.9–10.1, 10.2–12.7, and ≥12.8%). Quartiles of BP‐SD and BP‐CV were named the lowest, second, third, and highest, in ascending order from the lowest.

### Statistical Analysis

Data are presented as mean±SD or number (percentage). For comparison of patient characteristics and 2‐year event rates among the quartiles, trend analysis was performed using Cochran‐Armitage test for categorical variables or Jonckheere‐Terpstra test for continuous variables, as appropriate. Relation between 2 parameters was assessed by Pearson’s correlation coefficient analysis. Kaplan‐Meier curves among the quartiles were compared with a log‐rank test. Univariable and multivariable analyses using Cox proportional hazards models were performed to investigate the influence of BP variability on adverse events. Cox proportional hazards assumption was verified by the log‐log survival curve in all of the study outcomes. Risks of BP variability for adverse events in each quartile were expressed as hazard ratios (HRs) and 95% CIs with the lowest quartile as a reference. Explanatory variables for multivariable analysis were adopted from well‐known risk factors used in our previous subanalysis for hypertension and BP^7^; they included components of the CHA_2_DS_2_‐VASc score (congestive heart failure, hypertension, age ≥75 years, diabetes mellitus, history of stroke or transient ischemic attack, vascular disease [coronary artery disease], age 65–74 years, female sex),^9^ warfarin and antiplatelet use, type of AF, and BP measurement times (Model 1). Model 2 included variables of Model 1 plus BP‐end as explanatory variables based on the results of our previous subanalysis on BP^7^; and Model 3 included variables of Model 1 plus creatinine clearance (CrCl), body mass index (BMI), and hemoglobin levels according to the results from our previous reports on CrCl,^28^ BMI,^29^ and hemoglobin levels.^30^ The same analyses were performed using continuous values of the systolic and diastolic BP‐SD and BP‐CV as a sensitivity analysis. In addition, the predictive ability of systolic BP variability indices for adverse events determined by the area under the receiver operating characteristic curve was compared with that of systolic BP‐end using the DeLong’s test.^31^, ^32^ Two‐tailed *P* values of <0.05 were considered statistically significant. All statistical analyses were performed with SPSS software version 23.0 (IBM Corporation, Armonk, NY) and R version 3.5.2 (The R Foundation for Statistical Computing, Vienna, Austria).

## Results

Of the entire 7937 patients with AF enrolled in the J‐RHYTHM Registry,^24^ 421 (5.3%) with valvular AF^25^ were excluded and 110 (1.5%) were lost to follow‐up. Of the remaining 7406 patients with NVAF,^7^, ^33^ 180 (2.4%) patients with BP measurements <4 times during the follow‐up period were excluded. Consequently, 7226 patients (age, 69.7±9.9 years; men, 70.7%) were included in this subanalysis.

### Patient Characteristics and Medications

Clinical characteristics of 7226 patients are listed in Table S1. Sixty percent of the patients had hypertension, and BP was measured 14.6±5.0 times during the follow‐up period. Systolic and diastolic BP values at the time of enrollment were 126.0±16.1 mm Hg and 73.3±11.0 mm Hg (Table S1); and the mean values during the follow‐up period were 125.5±16.4 mm Hg and 72.8±11.1 mm Hg, respectively. Systolic BP‐SD and BP‐CV during the follow‐up period were 11.0±4.2 mm Hg and 8.8±3.2%, and diastolic BP‐SD and BP‐CV were 7.7±2.8 mm Hg and 10.7±4.1%, respectively (Table S1). These BP variability indices were not correlated with TTR in overall patients or those aged <70 years, but significantly correlated with TTR in patients aged ≥70 years and PT‐INR variability (Table S2).

Patient characteristics and medications in each systolic BP‐SD quartile are shown in Table 1. Age, systolic and diastolic BP, and prevalence of congestive heart failure, hypertension, age ≥75 years, diabetes mellitus, and prior stroke or transient ischemic attack showed significant trends across the quartiles, resulting in higher risk scores for the higher quartiles. In contrast, CrCl, BMI, and hemoglobin levels showed lower values in the highest quartile. Use of antihypertensive drugs and antiplatelet drugs was more prevalent in the higher quartiles, whereas frequency of warfarin use, PT‐INR, and TTR were comparable across the systolic BP‐SD quartiles (Table 1).

**Table 1:** Patient Characteristics and Medications in Each Systolic BP‐SD Quartile

	Lowest Quartile	Second Quartile	Third Quartile	Highest Quartile	*P* Value for Trend
Range of Systolic BP‐SD, mm Hg	<8.20	8.20–10.49	10.50–13.19	≥13.20	
Number of patients	1791	1819	1800	1816	
Age, y	67.9±10.2	69.1±9.9	70.2±9.4	71.6±9.7	<0.001
Sex, male	1328 (74.1)	1317 (72.4)	1245 (69.2)	1218 (67.1)	<0.001
Body mass index, kg/m^2^	23.6±3.3	23.9±3.8	23.6±3.6	23.5±4.9	0.028
Type of atrial fibrillation					
Paroxysmal	711 (39.7)	673 (37.0)	668 (37.1)	710 (39.1)	0.968
Persistent	262 (14.6)	244 (13.4)	280 (15.6)	270 (14.9)	
Permanent	818 (45.7)	902 (49.3)	852 (47.3)	836 (46.0)	
Comorbidities					
Coronary artery disease	183 (10.2)	190 (10.4)	171 (9.5)	211 (11.6)	0.309
Cardiomyopathy	160 (8.9)	152 (8.4)	157 (8.7)	151 (8.3)	0.614
HCM	61 (3.4)	64 (3.5)	66 (3.7)	67 (3.7)	0.610
DCM	99 (5.5)	88 (4.8)	91 (5.1)	84 (4.6)	0.280
Congenital heart disease	25 (1.4)	27 (1.5)	27 (1.5)	17 (0.9)	0.255
COPD	18 (1.0)	33 (1.8)	29 (1.6)	44 (2.4)	0.013
Hyperthyroidism	41 (1.7)	29 (1.6)	37 (2.1)	32 (1.8)	0.691
Risk factors for stroke					
Heart failure	422 (23.6)	507 (27.9)	502 (27.9)	567 (31.2)	<0.001
Hypertension	950 (53.0)	1044 (57.4)	1145 (63.8)	1239 (68.2)	<0.001
Age (≥75 y)	488 (27.2)	594 (32.7)	631 (35.1)	770 (42.4)	<0.001
Diabetes mellitus	278 (15.5)	333 (18.3)	334 (18.6)	381 (21.0)	<0.001
Stroke/TIA	211 (11.8)	225 (12.4)	265 (14.7)	290 (16.0)	<0.001
CHADS_2_ score	1.4±1.2	1.6±1.2	1.7±1.2	1.9±1.2	<0.001
CHA_2_DS_2_‐VASc score	2.5±1.5	2.7±1.6	2.9±1.6	3.2±1.6	<0.001
HAS‐BLED score	1.2±0.9	1.4±0.9	1.6±1.0	1.8±1.1	<0.001
BP measurement times	14.1±5.0	14.9±4.6	15.3±4.9	14.4±5.2	<0.001
Systolic BP, mm Hg	122.9±12.2	124.5±14.4	126.3±16.5	130.3±19.5	<0.001
SD, mm Hg	6.4±1.4	9.3±0.7	11.7±0.8	16.5±3.4	<0.001
CV, %	5.3±1.2	7.6±0.9	9.4±1.1	12.9±2.7	<0.001
Diastolic BP, mm Hg	73.0±9.4	72.8±10.2	73.4±11.2	74.2±12.9	<0.001
SD, mm Hg	5.8±2.1	7.1±2.1	8.1±2.3	9.8±3.1	<0.001
CV, %	8.1±3.1	9.9±3.2	11.2±3.4	13.5±4.5	<0.001
Heart rate/min	72.1±12.8	72.6±12.6	72.4±13.2	72.6±14.0	0.639
Creatinine clearance, mL/min	73.1±26.4	70.9±26.9	66.8±27.1	63.6±29.3	<0.001
Hemoglobin, g/dL	13.9±1.6	13.8±1.7	13.7±1.7	13.4±1.9	<0.001
Medications					
Warfarin	1528 (85.3)	1591 (87.5)	1583 (87.9)	1567 (86.3)	0.348
Dosage, mg/d	3.0±1.2	2.9±1.2	2.9±1.2	2.7±1.1	<0.001
PT‐INR	1.92±0.50	1.91±0.48	1.92±0.48	1.88±0.50	0.154
TTR*, %	58.7±29.9	60.1±28.8	60.6±28.3	58.9±28.2	0.795
Antiplatelet	426 (23.8)	449 (24.7)	490 (27.2)	517 (28.5)	<0.001
Aspirin	385 (21.5)	387 (21.3)	419 (23.3)	437 (24.1)	0.027
Others	77 (4.3)	93 (5.1)	111 (6.2)	139 (7.7)	<0.001
Warfarin+antiplatelet	288 (16.1)	327 (18.0)	355 (19.7)	359 (19.8)	0.002
ARB/ACE‐I	822 (45.9)	946 (52.0)	999 (55.5)	1093 (59.6)	<0.001
Antihypertensive drugs^†^	814 (45.4)	825 (45.4)	912 (50.7)	905 (49.8)	<0.001
Statins	402 (22.4)	451 (24.8)	469 (26.1)	435 (24.0)	0.205

Data are number of patients (%) or mean±SD. ACE‐I indicates angiotensin‐converting enzyme inhibitor; ARB, angiotensin II receptor blocker; BP, blood pressure; CHA_2_DS_2_‐VASc, additionally, vascular disease (coronary artery disease), age 65–74 years, and female sex; CHADS_2_, congestive heart failure, hypertension, age ≥75 years, diabetes mellitus, and history of stroke or TIA; COPD, chronic obstructive pulmonary disease; CV, coefficient of variation=SD/mean; DCM, dilated cardiomyopathy; HAS‐BLED, hypertension (systolic BP ≥140 mm Hg), abnormal renal/liver function, stroke, bleeding history or predisposition, labile INR (episodes of INR ≥3.5), elderly (age >65 years), drugs (use of antiplatelets)/alcohol concomitantly; HCM, hypertrophic cardiomyopathy; PT‐INR, prothrombin time international normalized ratio; SD, standard deviation; TIA, transient ischemic attack; and TTR, time in therapeutic range.

* Target PT‐INR was 2.0–3.0 (<70 years) or 1.6–2.6 (≥70 years).

^†^ Drugs other than ARB/ACE‐I.

### Influence of BP‐SD on Adverse Events

During the 2‐year follow‐up period, thromboembolism, major hemorrhage, and all‐cause death occurred in 110 (1.5%), 121 (1.7%), and 168 (2.3%) patients, respectively. Corresponding incidence rates of these events were 0.8, 0.8, and 1.2/100‐person years, respectively, during the follow‐up period of 14 580 person‐years.

Two‐year event rates in each systolic and diastolic BP‐SD quartile are summarized in Table 2. All event rates showed significant trends across systolic and diastolic BP‐SD quartiles (Table 2). Cumulative event‐free rates for all events were significantly different among BP‐SD quartiles (*P*<0.001 for all, by log‐rank test) in the Kaplan–Meier curves with the worst event‐free survival rates in the highest quartile (Figure).

**Table 2:** Two‐Year Event Rates in Each BP‐SD Quartile

Quartiles of Systolic BP‐SD (mm Hg)	Lowest Quartile (<8.20)	Second Quartile (8.20–10.49)	Third Quartile (10.50–13.19)	Highest Quartile (≥13.20)	*P* Value for Trend
Number of patients	1791	1819	1800	1816	
Thromboembolism	21 (1.2%)	20 (1.1%)	18 (1.0%)	51 (2.8%)	<0.001
Major hemorrhage	16 (0.9%)	24 (1.3%)	27 (1.5%)	54 (3.0%)	<0.001
All‐cause death	29 (1.6%)	25 (1.4%)	32 (1.8%)	82 (4.5%)	<0.001
Composite events*	66 (3.7%)	69 (3.8%)	77 (4.3%)	187 (10.3%)	<0.001

Quartiles of Diastolic BP‐SD (mm Hg)	Lowest Quartile (<5.80)	Second Quartile (5.80–7.29)	Third Quartile (7.30–9.29)	Highest Quartile (≥9.30)	*P* Value for trend
Number of patients	1707	1758	1866	1785	
Thromboembolism	27 (1.6%)	12 (0.7%)	28 (1.5%)	43 (2.4%)	0.012
Major hemorrhage	25 (1.4%)	20 (1.1%)	28 (1.5%)	48 (2.6%)	0.004
All‐cause death	29 (1.7%)	29 (1.6%)	32 (1.7%)	78 (4.3%)	<0.001
Composite events*	81 (4.7%)	61 (3.4%)	88 (4.6%)	169 (9.2%)	<0.001

Data are number of patients (%). BP indicates blood pressure; and SD, standard deviation.

* Thromboembolism, major hemorrhage, and all‐cause death.

HRs for thromboembolism, major hemorrhage, all‐cause death, and composite events were significantly high in the highest quartile compared with the lowest quartile in univariable unadjusted analysis (Table 3). This was also true in multivariable analysis adjusted for components of CHA_2_DS_2_‐VASc score, warfarin and antiplatelet use, type of AF, and BP measurement times (Model 1, Table 4); for variables of Model 1 plus BP‐end (Model 2, Table 5); and for variables of Model 1 plus CrCl, BMI, and hemoglobin levels (Model 3, Table 6). These results were consistent when baseline BP value was adopted for an adjusting covariate instead of hypertension (data not shown).

**Table 3:** Influence of BP‐SD on Adverse Events (Univariable Cox Proportional Hazards Analysis)

Adverse event	Thromboembolism		Major Hemorrhage		All‐Cause Death		Composite Events*	
	HR (95% CI)	*P* Value	HR (95% CI)	*P* Value	HR (95% CI)	*P*Value	HR (95% CI)	*P* Value
Systolic BP‐SD								
Lowest quartile (<8.20 mm Hg)	1.00 (reference)		1.00 (reference)		1.00 (reference)		1.00 (reference)	
Second quartile (8.20–10.49 mm Hg)	0.92 (0.50–1.64)	0.799	1.46 (0.77–2.74)	0.245	0.83 (0.49–1.42)	0.506	1.01 (0.72–1.42)	0.940
Third quartile (10.50–13.19 mm Hg)	0.85 (0.45–1.59)	0.602	1.67 (0.90–3.09)	0.106	1.09 (0.66–1.80)	0.743	1.15 (0.83–1.60)	0.403
Highest quartile (≥13.20 mm Hg)	2.46 (1.48–4.09)	0.001	3.42 (1.96–5.98)	<0.001	2.88 (1.89–4.40)	<0.001	2.88 (2.18–3.81)	<0.001
Diastolic BP‐SD								
Lowest quartile (<5.80 mm Hg)	1.00 (reference)		1.00 (reference)		1.00 (reference)		1.00 (reference)	
Second quartile (5.80–7.29 mm Hg)	0.42 (0.21–0.83)	0.013	0.76 (0.42–1.37)	0.760	0.95 (0.56–1.58)	0.830	0.71 (0.51–1.00)	0.047
Third quartile (7.30–9.29 mm Hg)	0.93 (0.55–1.57)	0.779	1.00 (0.58–1.72)	1.000	0.98 (0.59–1.62)	0.942	0.97 (0.72–1.31)	0.837
Highest quartile (≥9.30 mm Hg)	1.53 (0.95–2.47)	0.084	1.85 (1.14–2.99)	0.013	2.59 (1.69–3.97)	<0.001	2.01 (1.54–2.62)	<0.001

BP indicates blood pressure; HR, hazard ratio; and SD, standard deviation.

* Thromboembolism, major hemorrhage, and all‐cause death.

**Table 4:** Influence of BP‐SD on Adverse Events (Multivariable Cox Proportional Hazards Analysis, Model 1)

Adverse event	Thromboembolism		Major Hemorrhage		All‐Cause Death		Composite Events*	
	HR (95% CI)	*P* Value	HR (95% CI)	*P* Value	HR (95% CI)	*P* Value	HR (95% CI)	*P* Value
Systolic BP‐SD								
Lowest quartile (<8.20 mm Hg)	1.00 (reference)		1.00 (reference)		1.00 (reference)		1.00 (reference)	
Second quartile (8.20–10.49 mm Hg)	1.19 (0.64–2.21)	0.580	1.79 (0.95–3.39)	0.072	0.96 (0.56–1.65)	0.881	1.24 (0.88–1.74)	0.220
Third quartile (10.50–13.19 mm Hg)	1.11 (0.59–2.09)	0.752	2.01 (1.08–3.74)	0.028	1.19 (0.72–1.98)	0.496	1.37 (0.98–1.91)	0.063
Highest quartile (≥13.20 mm Hg)	2.24 (1.34–3.74)	0.002	2.97 (1.69–5.22)	<0.001	2.14 (1.39–3.29)	0.001	2.38 (1.79–3.17)	<0.001
Diastolic BP‐SD								
Lowest quartile (<5.80 mm Hg)	1.00 (reference)		1.00 (reference)		1.00 (reference)		1.00 (reference)	
Second quartile (5.80–7.29 mm Hg)	0.54 (0.27–1.08)	0.079	0.95 (0.52–1.72)	0.866	1.07 (0.64–1.80)	0.806	0.87 (0.62–1.21)	0.867
Third quartile (7.30–9.29 mm Hg)	1.29 (0.76–2.21)	0.351	1.31 (0.76–2.26)	0.334	1.06 (0.64–1.76)	0.831	1.20 (0.89–1.63)	0.238
Highest quartile (≥9.30 mm Hg)	1.22 (0.75–2.00)	0.426	1.54 (0.94–2.51)	0.085	1.82 (1.18–2.80)	0.007	1.55 (1.19–2.03)	0.001

Model 1: Adjusted for components of CHA_2_DS_2_‐VASc score, warfarin and antiplatelet use, type of atrial fibrillation, and BP measurement times. BP indicates blood pressure; HR, hazard ratio; and SD, standard deviation.

* Thromboembolism, major hemorrhage, and all‐cause death.

**Table 5:** Influence of BP‐SD on Adverse Events (Multivariable Cox Proportional Hazards Analysis, Model 2)

Adverse event	Thromboembolism		Major Hemorrhage		All‐Cause Death		Composite Events*	
	HR (95% CI)	*P* Value	HR (95% CI)	*P* Value	HR (95% CI)	*P* Value	HR (95% CI)	*P* Value
Systolic BP‐SD								
Lowest quartile (<8.20 mm Hg)	1.00 (reference)		1.00 (reference)		1.00 (reference)		1.00 (reference)	
Second quartile (8.20–10.49 mm Hg)	1.09 (0.56–2.13)	0.795	2.13 (1.08–4.21)	0.029	1.08 (0.59–1.94)	0.881	1.39 (0.96–2.00)	0.080
Third quartile (10.50–13.19 mm Hg)	1.10 (0.56–2.15)	0.782	2.36 (1.21–4.60)	0.011	1.12 (0.63–1.96)	0.706	1.52 (1.06–2.17)	0.022
Highest quartile (≥13.20 mm Hg)	1.90 (1.08–3.36)	0.027	3.40 (1.83–6.23)	<0.001	2.23 (1.37–3.64)	0.001	2.83 (2.07–3.86)	<0.001
Diastolic BP‐SD								
Lowest quartile (<5.80 mm Hg)	1.00 (reference)		1.00 (reference)		1.00 (reference)		1.00 (reference)	
Second quartile (5.80–7.29 mm Hg)	0.56 (0.26–1.18)	0.124	0.96 (0.52–1.79)	0.896	1.15 (0.64–2.01)	0.631	0.91 (0.64–1.31)	0.623
Third quartile (7.30–9.29 mm Hg)	1.43 (0.82–2.51)	0.210	1.30 (0.75–2.28)	0.351	0.97 (0.54–1.75)	0.922	1.23 (0.89–1.70)	0.219
Highest quartile (≥9.30 mm Hg)	1.40 (0.84–2.34)	0.199	1.58 (0.96–2.61)	0.074	2.05 (1.25–3.35)	0.004	1.76 (1.32–2.34)	<0.001

Model 2: Adjusted for variables of Model 1 plus BP at the closest time of the event or at the end of follow‐up. BP indicates blood pressure; HR, hazard ratio; and SD, standard deviation.

* Thromboembolism, major hemorrhage, and all‐cause death.

**Table 6:** Influence of BP‐SD on Adverse Events (Multivariable Cox Proportional Hazards Analysis, Model 3)

Adverse event	Thromboembolism		Major Hemorrhage		All‐Cause Death		Composite Events*	
	HR (95% CI)	*P* Value	HR (95% CI)	*P* Value	HR (95% CI)	*P* Value	HR (95% CI)	*P* Value
Systolic BP‐SD								
Lowest quartile (<8.20 mm Hg)	1.00 (reference)		1.00 (reference)		1.00 (reference)		1.00 (reference)	
Second quartile (8.20–10.49 mm Hg)	1.12 (0.57–2.18)	0.745	1.87 (0.91–3.84)	0.088	0.82 (0.43–1.57)	0.548	1.15 (0.78–1.69)	0.482
Third quartile (10.50–13.19 mm Hg)	0.83 (0.41–1.68)	0.603	2.07 (1.03–4.16)	0.041	1.12 (0.63–1.99)	0.707	1.27 (0.87–1.83)	0.213
Highest quartile (≥13.20 mm Hg)	2.00 (1.15–3.49)	0.015	2.60 (1.36–4.97)	0.004	1.85 (1.11–3.07)	0.018	2.12 (1.54–2.93)	<0.001
Diastolic BP‐SD								
Lowest quartile (<5.80 mm Hg)	1.00 (reference)		1.00 (reference)		1.00 (reference)		1.00 (reference)	
Second quartile (5.80–7.29 mm Hg)	0.56 (0.28–1.16)	0.118	0.84 (0.43–1.63)	0.597	0.96 (0.54–1.74)	0.904	0.78 (0.54–1.13)	0.188
Third quartile (7.30–9.29 mm Hg)	1.15 (0.64–2.08)	0.636	1.20 (0.66–2.20)	0.548	1.03 (0.59–1.81)	0.916	1.10 (0.79–1.54)	0.582
Highest quartile (≥9.30 mm Hg)	1.04 (0.62–1.75)	0.874	1.44 (0.84–2.47)	0.181	1.41 (0.88–2.27)	0.156	1.32 (0.98–1.76)	0.065

BP indicates blood pressure; HR, hazard ratio; and SD, standard deviation.

Model 3: Adjusted for variables of Model 1 plus creatinine clearance, body mass index, and hemoglobin levels (N=5774).

* Thromboembolism, major hemorrhage, and all‐cause death.

As for diastolic BP‐SD, HRs for major hemorrhage, all‐cause death, and composite events were significantly high in the highest quartile compared with the lowest quartile in the univariable unadjusted model (Table 3). In multivariable adjusted models, the significance for major hemorrhage disappeared in Models 1 and 2 (Tables 4 and 5), whereas that for all events disappeared in Model 3 (Table 6).

When BP‐SD was analyzed as a continuous variable, HRs (/1‐mm Hg increase) of systolic BP‐SD for all events were significantly high in univariable and multivariable analysis (Table 7); whereas HRs (/1‐mm Hg increase) of diastolic BP‐SD for adverse events became insignificant except for composite events in Model 2 (Table 7).

**Table 7:** Influence of BP‐SD as a Continuous Variable on Adverse Events (Cox Proportional Hazards Analysis)

Adverse event	Thromboembolism		Major Hemorrhage		All‐Cause Death		Composite Events*	
	HR (95% CI)	*P* Value	HR (95% CI)	*P* Value	HR (95% CI)	*P* Value	HR (95% CI)	*P* Value
Univariable								
Systolic BP‐SD (/1‐mm Hg increase)	1.13 (1.10–1.17)	<0.001	1.12 (1.09–1.16)	<0.001	1.11 (1.08–1.14)	<0.001	1.12 (1.10–1.14)	<0.001
Diastolic BP‐SD (/1‐mm Hg increase)	1.09 (1.03–1.16)	0.002	1.12 (1.06–1.18)	<0.001	1.15 (1.11–1.20)	<0.001	1.13 (1.10–1.16)	<0.001
Multivariable (Model 1)								
Systolic BP‐SD (/1‐mm Hg increase)	1.07 (1.05–1.10)	<0.001	1.08 (1.05–1.11)	<0.001	1.07 (1.04–1.10)	<0.001	1.07 (1.06–1.09)	<0.001
Diastolic BP‐SD (/1‐mm Hg increase)	1.03 (0.98–1.08)	0.286	1.06 (1.01–1.11)	0.026	1.07 (1.03–1.12)	0.001	1.14 (1.07–1.21)	<0.001
Multivariable (Model 2)								
Systolic BP‐SD (/1‐mm Hg increase)	1.04 (1.01–1.07)	0.020	1.07 (1.04–1.10)	<0.001	1.06 (1.03–1.09)	<0.001	1.08 (1.06–1.09)	<0.001
Diastolic BP‐SD (/1‐mm Hg increase)	1.04 (0.99–1.09)	0.116	1.05 (1.01–1.11)	0.031	1.06 (1.02–1.11)	0.004	1.06 (1.04–1.09)	<0.001
Multivariable (Model 3)								
Systolic BP‐SD (/1‐mm Hg increase)	1.07 (1.04–1.10)	<0.001	1.07 (1.03–1.10)	<0.001	1.06 (1.03–1.09)	<0.001	1.07 (1.05–1.09)	<0.001
Diastolic BP‐SD (/1‐mm Hg increase)	1.01 (0.96–1.07)	0.675	1.05 (0.99–1.11)	0.094	1.04 (1.00–1.09)	0.083	1.12 (1.04–1.21)	0.004

BP indicates blood pressure; HR, hazard ratio; and SD, standard deviation.

Model 1: adjusted for components of CHA_2_DS_2_‐VASc score, warfarin and antiplatelet use, type of atrial fibrillation, and BP measurement times.

Model 2: adjusted for variables of Model 1 plus BP at the time closest to the event or at the end of follow‐up.

Model 3: adjusted for variables of Model 1 plus creatinine clearance, body mass index, and hemoglobin level (N=5774).

* Thromboembolism, major hemorrhage, and all‐cause death.

The AUCs of BP‐SD for thromboembolism and all‐cause death were comparable with those of BP‐end, whereas the AUCs of BP‐SD for major hemorrhage and composite events were significantly larger than those of BP‐end (Table S3).

### Influence of BP‐CV on Adverse Events

When BP‐CV was used instead of BP‐SD, similar results were obtained. Event rates showed significant trends across BP‐CV quartiles (Table S4). HRs were significantly high for all events in the highest quartile of systolic and diastolic BP‐CV compared with the lowest quartile in univariable analysis (Table S5). Regarding systolic BP‐CV, this was true even in multivariable analysis (Tables S6–S8) as for systolic BP‐SD. In contrast, HR only for composite events in the highest quartile of diastolic BP‐CV remained significant in Model 3 (Table S8).

When BP‐CV was analyzed as a continuous variable, HRs (/1% increase) of systolic BP‐CV were significantly high for all events in multivariable analysis as well as in unadjusted analysis (Table S9), whereas HRs (/1% increase) of diastolic BP‐CV for all‐cause death and composite events were significantly high in Model 3 (Table S9).

The AUCs of BP‐CV for thromboembolism and all‐cause death were comparable with those of BP‐end, whereas the AUCs of BP‐CV for major hemorrhage and composite events were significantly larger than those of BP‐end (Table S3).

## Discussion

The major findings of this study were as follows. First, age, BP, and prevalence of comorbidities showed significant trends across the quartiles of systolic BP variability. Second, the highest quartile of systolic BP‐SD of ≥13.20 mm Hg was independently associated with the increased incidence of all adverse events, but that of diastolic BP‐SD was not. Third, these findings were consistent when using BP‐CV instead of BP‐SD.

### Impact of BP Variability on Adverse Events in Patients Without AF

In 1997, Grove et al.^34^ reported that BP visit‐to‐visit variability, an index of long‐term BP variability,^16^ was associated with coronary artery disease. After more than a decade, in 2010, Rothwell et al. indicated limited impact of usual BP values on adverse prognosis, but stressed impact of BP variability, instability, and episodic hypertension on adverse outcomes.^35^ In addition, they demonstrated that BP visit‐to‐visit variability was a strong risk factor for stroke, independent of mean BP values.^17^ Subsequently, several investigators scrutinized this issue, and systematic reviews and meta‐analyses showed that BP visit‐to‐visit variability was an independent predictor of incident cardiovascular diseases, coronary artery disease, stroke, and all‐cause and cardiovascular death.^18^, ^36^, ^37^ However, these studies did not focus on patients with NVAF.^17^, ^18^, ^34^, ^35^, ^36^, ^37^

### Impact of BP Variability on Adverse Events in Patients With AF

There have been few studies on association of BP variability with clinical outcome in patients with NVAF. In 2017, Proietti et al.^22^ reported the association between systolic BP visit‐to‐visit variability and major adverse outcomes in patents with AF in a post hoc analysis of the AFFIRM Study.^38^ They demonstrated that patients in the highest quartile of systolic BP‐SD were older and more likely to be female, and had higher systolic and diastolic BP values, prevalence of comorbidities, and CHA_2_DS_2_‐VASc score. Additionally, the third and highest quartiles of systolic BP‐SD were independently associated with a higher risk for stroke (HR, 1.85, *P*=0.042 and HR, 2.33, *P*=0.004, respectively) as well as major hemorrhage (HR, 1.92, *P*=0.009 and HR, 2.88, *P*<0.001, respectively). Patients in the highest quartile also had a higher risk for all‐cause death (HR, 1.38, *P*=0.048).^22^ Diastolic BP‐SD was not analyzed in that post hoc analysis.^22^

Our post hoc analysis showed results similar to those in the AFFIRM post hoc analysis.^22^ That is, clinical characteristics including age, sex, prevalence of comorbidities, and risk scores showed significant trends across the systolic BP‐SD quartiles. The highest quartile of systolic BP‐SD was significantly associated with increased risk of thromboembolism, major hemorrhage, and all‐cause death even after adjusting for multiple confounding factors (Tables 4, 5, 6). This was also true when systolic BP‐CV was used instead of BP‐SD (Tables S6–S8). In addition, these results were confirmed using BP‐SD and BP‐CV as a continuous variable (Table 7 and Table S9). In contrast, diastolic BP variability was not associated with adverse outcome events in multivariable analysis (Model 3, Table 6), except for diastolic BP‐CV for composite events (Model 3, Table S8).

Since our previous report revealed that BP‐end, not BP at baseline, was independently associated with the incidence of both thromboembolism and major hemorrhage,^7^ we confirmed whether BP variability was independently associated with adverse events in the BP‐end‐adjusted model (Model 2). The results in this model were consistent with those of Model 1, indicating that the BP variability was independent of the BP‐end (Table 5 and Table S7). In addition, the AUCs of both systolic BP‐SD and BP‐CV for major hemorrhage and composite events were significantly higher than those of the systolic BP‐end (Table S3). These findings indicate that the systolic BP variability is superior to the simple systolic BP‐end as a predictor of major hemorrhage and composite events.

As shown in Table 1, several comorbidities and clinical variables showed significant trends across the systolic BP‐SD quartiles, indicating that patients with larger systolic BP‐SD had poorer clinical conditions and higher risk of adverse events. Therefore, HRs were adjusted for these variables to remove the confounding effects of these factors in Model 3. Nevertheless, the association between the systolic BP variability and adverse events was still significant even after adjusting for these variables. Thus, the present results indicated that the systolic BP variability was an independent risk factor for adverse events, even though it is difficult to adjust completely unmeasured variables and unknown confounding factors in a post hoc analysis of the observational study.

Several mechanisms have been proposed for the association of increased BP variability with adverse cardiovascular events.^16^, ^19^ Among them were poor adherence to drugs,^39^ poor quality of care,^22^ atherosclerosis,^40^ increased intima‐media thickness,^41^ and endothelial dysfunction.^42^, ^43^ Unfortunately, our observational study was not designed to determine the mechanism underlying for the increased BP variability itself and for the association between increased BP variability and adverse events.

### Relation Between BP Variability and Quality of Anticoagulation Therapy

In subanalysis of the AFFIRM Study,^22^ a significant inverse linear association was observed between the systolic BP‐SD and the quality of anticoagulation control evaluated by TTR. This was not the case in our study, which showed that TTR did not show significant trend across the systolic BP‐SD quartiles (Table 1) and the BP variability indices were not correlated with TTR in overall patients (Table S2). Difference in the quality of BP control and adherence to treatment might be associated with the different result. BP control in this study was ≈10 mm Hg better than that in the AFFIRM Study,^22^ resulting in the cutoff BP‐SD values of quartiles in the AFFIRM Study (10.09, 13.86, and 17.34 mm Hg)^22^ being higher than those in this study (8.20, 10.50, and 13.20 mm Hg). Comparable TTR values across the systolic BP‐SD quartiles might suggest comparable adherence to anticoagulation with warfarin across the quartiles in this study. Adherence to treatment, however, could hardly be compared between the 2 studies.

On the other hand, when evaluating separately in patients aged <70 years and ≥70 years since the Japanese guidelines^27^ recommended the age‐specific target PT‐INR of 2.0 to 3.0 (<70 years) and 1.6 to 2.6 (≥70 years), BP variability indices were significantly correlated with TTR in patients aged ≥70 years (Table S2). In addition, BP variability indices were also significantly correlated with PT‐INR variability, both PT‐INR‐SD and ‐CV (Table S2), which are independent of the age‐specific target PT‐INR. Thus, these results would be partially consistent with those in the AFFIRM Study.^22^

### Limitations

This study had several limitations. First, it was a post hoc analysis of data from the J‐RHYTHM Registry^24^, ^33^ and was therefore hypothesis‐generating in nature. Second, study subjects were recruited from only 158 institutions in Japan and most of the participating physicians specialized in cardiology and in the management of cardiac arrhythmias. Therefore, these results may not be generalizable to the overall Japanese population with NVAF. In addition, since all study subjects were Japanese in this study, these data may not necessarily be applicable to other racial/ethnic groups. Third, BP measurement methods were not standardized. BP values were obtained by the auscultatory method or an automated sphygmomanometer, as appropriate for daily clinical practice in each institution. Beat‐to‐beat variation of BP because of irregular heartbeats of AF might have affected BP measurement. Particularly, AF tachycardia could have hampered precise BP measurement; however, heart rate did not show significant trend across the quartiles in this study (Table 1). Fourth, changes in antihypertensive drugs and dosage, and adherence to drugs during the follow‐up period were not considered in the analysis. Fifth, Model 3 of the multivariable analysis excluded 1452 patients because of lack of data on CrCl, BMI, or hemoglobin levels. However, this exclusion might not have affected the results significantly because 2‐year incidence rates of all adverse events were comparable between patients included in and excluded from Model 3 (Table S10). Finally, only the association of BP variability with adverse events was analyzed in this study, and any causality could not be determined. Moreover, the mechanisms of increased BP variability were not determined. Patients in the highest BP‐SD quartile were associated with higher age and higher prevalence of comorbidities, a finding suggestive of higher BP variability being a simple marker of high‐risk clinical profile. It is unknown whether the BP variability would be a treatment target in patients with AF to improve clinical outcomes.

## Conclusions

Systolic BP visit‐to‐visit variability was an independent risk for thromboembolism, major hemorrhage, and all‐cause death in patients with NVAF. Overall quality of anticoagulation control with warfarin was not associated with BP variability. Further studies are needed to clarify the causality between BP variability and adverse clinical outcomes in patients with NVAF.

## Appendix

The following physicians participated in the J‐RHYTHM Registry: Executive Committee: H. Inoue, K. Okumura, H. Atarashi, and T. Yamashita. Local Executive Committee: M. Sakurai, Y. Kawamura (Hokkaido); K. Okumura, I. Kubota (Tohoku); Y. Kaneko, K. Matsumoto (North Kanto); S. Ogawa, H. Atarashi, T. Yamashita (South Kanto); H. Inoue, Y. Aizawa (Hokuetsu); I. Kodama, E. Watanabe (Chubu); Y. Koretsune, Y. Okuyama (Kansai); A. Shimizu, O. Igawa (Chugoku); S. Bando, M. Fukatani (Shikoku); T. Saikawa, A. Chishaki (Kyushu). Statistical Advisor: H. Origasa. Participating Investigators: N. Kato, K. Kanda, J. Kato, H. Obata, M. Aoki, H. Honda (Hokkaido); Y. Konta, T. Hatayama, Y. Abe, K. Terata, T. Yagi, A. Ishida, T. Komatsu, H. Tachibana, H. Suzuki, Y. Kamiyama, T. Watanabe, M. Oguma, M. Itoh, O. Hirono, Y. Tsunoda, K. Ikeda, T. Kanaya, K. Sakurai, H. Sukekawa, S. Nakada (Tohoku); T. Itoh, S. Tange, M. Manita, M. Ohta, H. Eguma, R. Kato, Y. Endo, T. Ogino, M. Yamazaki, H. Kanki, M. Uchida, S. Miyanaga, K. Shibayama, N. Toratani, T. Kojima, M. Ichikawa, M. Saito, Y. Umeda, T. Sawanobori, H. Sohara, S. Okubo, T. Okubo, T. Tokunaga, O. Kuboyama, H. Ito, Y. Kitahara (North Kanto); K. Sagara, T. Satoh, E. Kodani, K. Sugi, Y. Kobayashi, Y. Higashi, T. Katoh, Y. Hirayama, N. Matsumoto, M. Takano, T. Ikeda, S. Yusu, S. Niwano, Y. Nakazato, Y. Kawano, M. Sumiyoshi, N. Hagiwara, K. Murasaki, H. Mitamura, S. Nakagawa, K. Okishige, K. Azegami, H. Aoyagi, K. Sugiyama, M. Nishizaki, N. Yamawake, I. Watanabe, K. Ohkubo, H. Sakurada, S. Fukamizu, M. Suzuki, W. Nagahori, T. Nakamura, Y. Murakawa, N. Hayami, K. Yoshioka, M. Amino, K. Hirao, A. Yagishita, K. Ajiki, K. Fujiu, Y. Imai, A. Yamashina, T. Ishiyama (South Kanto); M. Sakabe, K. Nishida, H. Asanoi, H. Ueno, J. D. Lee, Y. Mitsuke, H. Furushima, K. Ebe, M. Tagawa, M. Sato, M. Morikawa (Hokuetsu); K. Yamashiro, K. Takami, T. Ozawa, M. Watarai, M. Yamauchi, H. Kamiya, H. Hirayama, Y. Yoshida, T. Murohara, Y. Inden, H. Osanai, N. Ohte, T. Goto, I. Morishima, T. Yamamoto, E. Fujii, M. Senga, H. Hayashi, T. Urushida, Y. Takada, R. Kato, N. Tsuboi, T. Noda, T. Hirose, T. Onodera, S. Kageyama, T. Osaka, T. Tomita, K. Shimada, M. Nomura, H. Izawa, A. Sugiura, T. Arakawa, K. Kimura (Chubu); T. Mine, T. Makita, H. Mizuno, A. Kobori, T. Haruna, M. Takagi, T. Watanabe, N. Tanaka, H. Shimizu, T. Kurita, K. Motoki, N. Takeda, Y. Kijima, M. Ito, A. Nakata, Y. Ueda, A. Hirata, S. Kamakura, K. Satomi, T. Noda, Y. Yamada (Kansai); Y. Yoshiga, H. Ogawa, M. Kimura, T. Hayano, T. Kinbara, H. Tatsuno, M. Harada, K. Kusano, M. Adachi, A. Yano, M. Sawaguchi, J. Yamasaki, T. Matsuura, Y. Tanaka, H. Moritani, T. Maki, S. Okada, M. Takechi, T. Hamada (Chugoku); A. Nishikado, Y. Takagi, I. Matsumoto, T. Yamamoto, T. Soeki, Y. Doi, M. Okawa, H. Seo, S. Kitamura, K. Yamamoto, M. Akizawa, N. Kaname (Shikoku); S. Ando, S. Narita, T. Nakamura, T. Inou, Y. Fukuizumi, K. Saku, M. Ogawa, Y. Urabe, M. Ikeuchi, S. Harada, H. Yamabe, Y. Imamura, Y. Yamanouchi, K. Sadamatsu, K. Yoshida, T. Kubota, N. Takahashi, N. Makino, Y. Higuchi, T. Ooie, T. Iwao, K. Kitamura, T. Imamura, K. Maemura, N. Komiya, M. Hayano, H. Yoshida, K. Yamashiro, K. Kumagai (Kyushu).

## Sources of Funding

The J‐RHYTHM Registry is registered at the University Hospital Medicine Information Network (UMIN) Clinical Trials Registry (UMIN000001569) and was supported by a grant from the Japan Heart Foundation (12080025). This research was partially supported by the Practical Research Project for Life‐Style related Diseases including Cardiovascular Diseases and Diabetes Mellitus from the Japan Agency for Medical Research and Development (AMED) (19ek0210082h0003).

## Disclosures

Dr. Kodani received remuneration from Daiichi‐Sankyo, Bristol‐Myers Squibb, and Ono Pharmaceutical; Dr. Inoue received remuneration from Daiichi‐Sankyo, Bayer Healthcare, Boehringer Ingelheim, and Bristol‐Myers Squibb; Dr. Atarashi received remuneration from Daiichi‐Sankyo; Dr. Okumura received research funding from Boehringer Ingelheim and Daiichi‐Sankyo and remuneration from Boehringer Ingelheim, Bayer Healthcare, Daiichi‐Sankyo, and Pfizer; Dr. Yamashita received research funding from Daiichi‐Sankyo, Bayer Healthcare, and Bristol‐Meyers Squibb and remuneration from Daiichi‐Sankyo, Pfizer, Bayer Healthcare, Bristol‐Myers Squibb, Toa Eiyo, and Ono Pharmaceutical; and Dr. Origasa received remuneration from Daiichi‐Sankyo and Bayer Healthcare.

**Figure 1:**
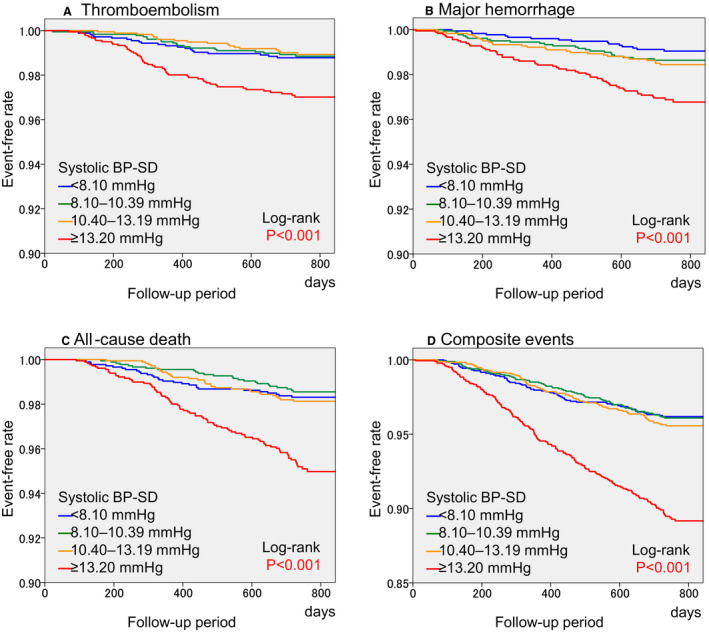
Kaplan–Meier curves for thromboembolism (**A**), major hemorrhage (**B**), all‐cause death (**C**), and composite events (**D**). *P* values: comparison among systolic BP‐SD quartiles by log‐rank test. BP indicates blood pressure; and SD, standard deviation.

## Supporting information


**Tables S1–S10**
