# Supplementary material for: Impact of Blood Pressure Visit‐to‐Visit Variability on Adverse Events in Patients With Nonvalvular Atrial Fibrillation: Subanalysis of the J‐RHYTHM Registry
Source: J Am Heart Assoc. 2020 Dec 29;10(1):e018585. doi: 10.1161/JAHA.120.018585 (PMC7955501; doi:10.1161/JAHA.120.018585)
Supplement: Supplementary file 1 — Tables S1–S10 [file JAH3-10-e018585-s001.pdf]

Supplemental Material

Table S1. Patient characteristics and medications.

Number of patients	7226
Age, years	69.7±9.9
Sex, male	5108 (70.7)
Body mass index, kg/m^2 (n=6242)	23.6±4.0
Type of atrial fibrillation	
Paroxysmal	2762 (38.2)
Persistent	1056 (14.6)
Permanent	3408 (47.2)
Comorbidities	
Coronary artery disease	755 (10.4)
Cardiomyopathy	620 (8.6)
HCM	258 (3.6)
DCM	362 (5.0)
Congenital heart disease	96 (1.3)
COPD	124 (1.7)
Hyperthyroidism	129 (1.8)
Risk factors for stroke	
Heart failure	1998 (27.7)
Hypertension	4378 (60.6)
Age (≥75 years)	2483 (34.4)
Diabetes mellitus	1326 (18.4)
Stroke/TIA	991 (13.7)
$CHADS_2$ score	1.7±1.2
CHA_2DS_2-VASc score	2.8±1.6
HAS-BLED score (n=6846)	1.5±1.0
BP measurement times	14.6±5.0
Systolic BP, mmHg	126.0±16.1
SD, mmHg	11.0±4.2
CV, %	8.8±3.2
Diastolic BP, mmHg	73.3±11.0
SD, mmHg	7.7±2.8
CV, %	10.7±4.1
Heart rate, /min	72.4±13.2
Creatinine clearance, mL/min (n=5925)	68.5±27.7
Hemoglobin, g/dL (n=6398)	13.7±1.7
Medications	
Warfarin	6269 (86.8)
Dosage, mg/day (n=6269)	2.9±1.2
PT-INR (n=6269)	1.91±0.49
TTR*, % (n=5934)	59.6±28.8
Antiplatelet	1882 (26.0)
Aspirin	1928 (22.5)
Others	420 (5.8)
Warfarin+antiplatelet	1329 (18.4)
ARB/ACE-I	3850 (53.3)
Antihypertensive drugs†	3456 (47.8)
Statins	1757 (24.3)

Data are number of patients (%) or mean±SD

BP, blood pressure; SD, standard deviation; HCM, hypertrophic cardiomyopathy; DCM, dilated cardiomyopathy; COPD, chronic obstructive pulmonary disease; TIA, transient ischemic attack; $CHADS_2$, congestive heart failure, hypertension, age ≥75 years, diabetes mellitus, and history of stroke or TIA; CHA_2DS_2-VASc, additionally, vascular disease (coronary artery disease), age 65–74 years, and female sex; HAS-BLED, hypertension (systolic BP ≥140 mmHg), abnormal renal/liver function, stroke, bleeding history or predisposition, labile INR (episodes of INR ≥3.5), elderly (age >65 years), drugs (use of antiplatelets)/alcohol concomitantly; CV, coefficient of variation=SD/mean; PT-INR, prothrombin time international normalized ratio; TTR, time in therapeutic range; ARB, angiotensin II receptor blacker; ACE-I, angiotensin converting enzyme inhibitor.

* Target PT-INR was 2.0–3.0 (<70 years) or 1.6–2.6 (≥70 years).

† Drugs other than ARB/ACE-I.

Table S2. Relation between BP variability and anticoagulation therapy.

	Quality of anticoagulation therapy						PT-INR variability			
	TTR* (Overall)		TTR* (<70 years)		TTR* (≥70 years)		PT-INR-SD		PT-INR-CV	
**BP variability indices**	**r †**	***P*-Value**	**r †**	***P*-Value**	**r †**	***P*-Value**	**r †**	***P*-Value**	**r †**	***P*-Value**
**Systolic BP-SD**	0.008	0.562	0.005	0.779	-0.112	<0.001	0.082	<0.001	0.099	<0.001
**Diastolic BP-SD**	–0.015	0.249	-0.023	0.238	-0.080	<0.001	0.093	<0.001	0.103	<0.001
**Systolic BP-CV**	0.002	0.899	0.009	0.634	-0.119	<0.001	0.107	<0.001	0.119	<0.001
**Diastolic BP-CV**	0.008	0.556	-0.027	0.169	-0.092	<0.001	0.113	<0.001	0.119	<0.001

* Target PT-INR was 2.0–3.0 (<70 years) or 1.6–2.6 (≥70 years).

† Pearson's correlation coefficient.

BP, blood pressure; TTR, time in therapeutic range; PT-INR, prothrombin time international normalized ratio; SD, standard deviation; CV, coefficient of variation=SD/mean.

Table S3. Predictive ability of systolic BP indices for adverse events.

	Thromboembolism		Major hemorrhage		All-cause death		Composite events*	
	AUC (95% CI)	*P*-Value†	AUC (95% CI)	*P*-Value†	AUC (95% CI)	*P*-Value†	AUC (95% CI)	*P*-Value†
Systolic BP-end‡	0.67 (0.60–0.73)	-	0.55 (0.48–0.61)	-	0.70 (0.64–0.75)	-	0.51 (0.47–0.55)	-
Systolic BP-SD	0.64 (0.58–0.71)	0.551	0.65 (0.60–0.70)	0.012	0.64 (0.59–0.69)	0.152	0.65 (0.62–0.68)	<0.001
Systolic BP-CV	0.64 (0.58–0.70)	0.475	0.64 (0.58–0.69)	0.041	0.69 (0.64–0.74)	0.758	0.66 (0.63–0.69)	<0.001

* Thromboembolism, major hemorrhage, and all-cause death.

† Compared with the systolic BP-end by the DeLong's test.

‡ BP values at the time closest to the event or at the end of follow-up.

BP, blood pressure; SD, Standard deviation; CV, coefficient of variation=SD/mean; AUC, area under the receiver operating characteristic (ROC) curve; CI, confidence interval.

Table S4. Two-year event rates in each BP-CV quartile.

Quartiles of systolic BP-CV (%)	Lowest quartile (<6.6)	Second quartile (6.6–8.3)	Third quartile (8.4–10.4)	Highest quartile (≥10.5)	*P*-Value for trend
Number of patients	1769	1819	1779	1859	
**Thromboembolism**	21 (1.2%)	22 (1.2%)	16 (0.9%)	51 (2.7%)	<0.001
**Major hemorrhage**	17 (1.0%)	24 (1.3%)	29 (1.6%)	51 (2.7%)	<0.001
**All-cause death**	25 (1.4%)	19 (1.0%)	38 (2.1%)	86 (4.6%)	<0.001
**Composite events***	63 (3.6%)	65 (3.6%)	83 (4.7%)	188 (10.1%)	<0.001
**Quartiles of diastolic BP-CV (%)**	**Lowest quartile (<7.9)**	**Second quartile (7.9–10.1)**	**Third quartile (10.2–12.7)**	**Highest quartile (≥12.8)**	***P*-Value for trend**
Number of patients†	1738	1868	1777	1843	
**Thromboembolism**	21 (1.2%)	21 (1.1%)	24 (1.4%)	44 (2.4%)	0.003
**Major hemorrhage**	26 (1.5%)	22 (1.2%)	25 (1.4%)	48 (2.6%)	0.007
**All-cause death**	24 (1.4%)	22 (1.2%)	34 (1.9%)	88 (4.8%)	<0.001
**Composite events***	71 (4.1%)	65 (3.5%)	83 (4.7%)	180 (9.8%)	<0.001

* Thromboembolism, major hemorrhage, and all-cause death.

Data are number of patients (%).

BP, blood pressure; CV, coefficient of variation=standard deviation/mean.

Table S5. Influence of BP-CV on adverse events (Univariable Cox proportional hazards analysis).

	Thromboembolism		Major hemorrhage		All-cause death		Composite events*	
	HR (95% CI)	*P*-Value	HR (95% CI)	*P*-Value	HR (95% CI)	*P*-Value	HR (95% CI)	*P*-Value
**Systolic BP-CV**								
Lowest quartile (<6.6%)	1.00 (reference)		1.00 (reference)		1.00 (reference)		1.00 (reference)	
Second quartile (6.6–8.3%)	1.01 (0.55–1.83)	0.987	1.35 (0.73–2.52)	0.339	0.73 (0.40–1.32)	0.295	0.99 (0.70–1.40)	0.948
Third quartile (8.4–10.4%)	0.75 (0.39–1.44)	0.392	1.68 (0.93–3.07)	0.088	1.50 (0.91–2.48)	0.116	1.30 (0.94–1.81)	0.116
Highest quartile (≥10.5%)	2.38 (1.43–3.95)	0.001	2.94 (1.70–5.08)	<0.001	3.39 (2.17–5.29)	<0.001	2.93 (2.20–3.90)	<0.001
**Diastolic BP-CV**								
Lowest quartile (<7.9%)	1.00 (reference)		1.00 (reference)		1.00 (reference)		1.00 (reference)	
Second quartile (7.9–10.1%)	0.91 (0.50–1.67)	0.765	0.77 (0.44–1.36)	0.370	0.83 (0.47–1.49)	0.536	0.83 (0.59–1.17)	0.289
Third quartile (10.2–12.7%)	1.10 (0.61–1.97)	0.752	0.92 (0.53–1.60)	0.779	1.36 (0.81–2.29)	0.250	1.12 (0.82–1.54)	0.473
Highest quartile (≥12.8%)	2.01 (1.19–3.37)	0.009	1.77 (1.10–2.85)	0.019	3.53 (2.25–5.55)	<0.001	2.43 (1.85–3.20)	<0.001

* Thromboembolism, major hemorrhage, and all-cause death.

BP, blood pressure; CV, coefficient of variation=standard deviation/mean; HR, hazard ratio; CI, confidence interval.

Table S6. Influence of BP-CV on adverse events (Multivariable Cox proportional hazards analysis, Model 1).

	Thromboembolism		Major hemorrhage		All-cause death		Composite events*	
	HR (95% CI)	*P*-Value	HR (95% CI)	*P*-Value	HR (95% CI)	*P*-Value	HR (95% CI)	*P*-Value
**Systolic BP-CV**								
Lowest quartile (<6.6%)	1.00 (reference)		1.00 (reference)		1.00 (reference)		1.00 (reference)	
Second quartile (6.6–8.3%)	0.98 (0.54–1.79)	0.955	1.26 (0.68–2.35)	0.466	0.60 (0.33–1.08)	0.089	0.90 (0.64–1.27)	0.549
Third quartile (8.4–10.4%)	0.72 (0.38–1.38)	0.324	1.54 (0.84–2.81)	0.160	1.10 (0.66–1.84)	0.712	1.13 (0.82–1.57)	0.459
Highest quartile (≥10.5%)	2.12 (1.27–3.56)	0.004	2.53 (1.45–4.42)	0.001	1.96 (1.24–3.10)	0.004	2.23 (1.67–2.99)	<0.001
**Diastolic BP-CV**								
Lowest quartile (<7.9%)	1.00 (reference)		1.00 (reference)		1.00 (reference)		1.00 (reference)	
Second quartile (7.9–10.1%)	1.19 (0.65–2.20)	0.568	0.98 (0.55–1.73)	0.935	0.93 (0.52–1.66)	0.792	1.01 (0.72–1.41)	0.964
Third quartile (10.2–12.7%)	1.56 (0.86–2.83)	0.142	1.18 (0.68–2.06)	0.555	1.45 (0.86–2.47)	0.167	1.39 (1.01–1.92)	0.045
Highest quartile (≥12.8%)	1.60 (0.94–2.73)	0.084	1.44 (0.87–2.35)	0.141	2.11 (1.33–3.35)	0.002	1.74 (1.32–2.31)	<0.001

* Thromboembolism, major hemorrhage, and all-cause death.
Model 1: Adjusted for components of CHA_2DS_2-VASc score, warfarin and antiplatelet use, type of atrial fibrillation, and BP measurement times.
BP, blood pressure; CV, coefficient of variation=standard deviation/mean; HR, hazard ratio; CI, confidence interval.

Table S7. Influence of BP-CV on adverse events (Multivariable Cox proportional hazards analysis, Model 2).

	Thromboembolism		Major hemorrhage		All-cause death		Composite events*	
	HR (95% CI)	*P*-Value	HR (95% CI)	*P*-Value	HR (95% CI)	*P*-Value	HR (95% CI)	*P*-Value
**Systolic BP-CV**								
Lowest quartile (<6.6%)	1.00 (reference)		1.00 (reference)		1.00 (reference)		1.00 (reference)	
Second quartile (6.6–8.3%)	1.38 (0.72–2.62)	0.333	1.87 (0.96–3.62)	0.066	0.79 (0.41–1.54)	0.497	1.31 (0.90–1.90)	0.160
Third quartile (8.4–10.4%)	1.04 (0.52–2.11)	0.904	2.60 (1.37–4.95)	0.004	1.41 (0.78–2.55)	0.258	1.68 (1.17–2.42)	0.005
Highest quartile (≥10.5%)	2.30 (1.32–4.00)	0.003	3.11 (1.71–5.67)	<0.001	2.21 (1.30–3.75)	0.003	2.92 (2.13–4.01)	<0.001
**Diastolic BP-CV**								
Lowest quartile (<7.9%)	1.00 (reference)		1.00 (reference)		1.00 (reference)		1.00 (reference)	
Second quartile (7.9–10.1%)	1.38 (0.72–2.66)	0.336	1.01 (0.56–1.84)	0.969	0.66 (0.34–1.29)	0.225	0.99 (0.68–1.42)	0.945
Third quartile (10.2–12.7%)	1.85 (0.99–3.46)	0.053	1.22 (0.69–2.17)	0.496	1.11 (0.61–2.00)	0.738	1.37 (0.97–1.93)	0.071
Highest quartile (≥12.8%)	1.95 (1.10–3.45)	0.022	1.60 (0.97–2.66)	0.067	1.80 (1.08–2.98)	0.023	1.90 (1.41–2.55)	<0.001

* Thromboembolism, major hemorrhage, and all-cause death.
Model 2: Adjusted for variables of Model 1 plus BP at the closest time of the event or at the end of follow-up.
BP, blood pressure; SD, standard deviation; HR. hazard ratio; CI, confidence interval.

Table S8. Influence of BP-CV on adverse events (Multivariable Cox proportional hazards analysis, Model 3).

	Thromboembolism		Major hemorrhage		All-cause death		Composite events*	
	HR (95% CI)	*P*-Value	HR (95% CI)	*P*-Value	HR (95% CI)	*P*-Value	HR (95% CI)	*P*-Value
**Systolic BP-CV**								
Lowest quartile (<6.6%)	1.00 (reference)		1.00 (reference)		1.00 (reference)		1.00 (reference)	
Second quartile (6.6–8.3%)	1.34 (0.69–2.62)	0.393	1.53 (0.75–3.11)	0.243	0.69 (0.34–1.42)	0.314	1.09 (0.73–1.63)	0.668
Third quartile (8.4–10.4%)	0.99 (0.49–2.01)	0.987	2.03 (1.03–3.99)	0.040	1.49 (0.83–2.69)	0.181	1.48 (1.02–2.14)	0.037
Highest quartile (≥10.5%)	2.11 (1.19–3.73)	0.010	2.31 (1.22–4.34)	0.010	1.93 (1.13–3.31)	0.017	2.14 (1.53–2.98)	<0.001
**Diastolic BP-CV**								
Lowest quartile (<7.9%)	1.00 (reference)		1.00 (reference)		1.00 (reference)		1.00 (reference)	
Second quartile (7.9–10.1%)	1.21 (0.64–2.29)	0.561	1.01 (0.53–1.92)	0.970	0.88 (0.46–1.68)	0.694	1.00 (0.69–1.45)	0.997
Third quartile (10.2–12.7%)	1.38 (0.73–2.63)	0.327	1.32 (0.71–2.43)	0.378	1.35 (0.75–2.43)	0.315	1.35 (0.95–1.92)	0.096
Highest quartile (≥12.8%)	1.39 (0.79–2.44)	0.257	1.35 (0.77–2.35)	0.293	1.56 (0.94–2.61)	0.087	1.46 (1.07–2.00)	0.017

* Thromboembolism, major hemorrhage, and all-cause death.

Model 3: Adjusted for variables of Model 1 plus creatinine clearance, body mass index, and hemoglobin level (N=5774).

BP, blood pressure; CV, coefficient of variation=standard deviation/mean; HR, hazard ratio; CI, confidence interval.

Table S9. Influence of BP-CV as a continuous variable on adverse events (Cox proportional hazards analysis).

	Thromboembolism		Major hemorrhage		All-cause death		Composite events*	
	HR (95% CI)	*P*-Value	HR (95% CI)	*P*-Value	HR (95% CI)	*P*-Value	HR (95% CI)	*P*-Value
**Univariable**								
Systolic BP-CV (/1% increase)	1.17 (1.13–1.22)	<0.001	1.16 (1.11–1.21)	<0.001	1.19 (1.16–1.23)	<0.001	1.78 (1.15–1.20)	<0.001
Diastolic BP-CV (/1% increase)	1.06 (1.02–1.10)	0.005	1.07 (1.04–1.11)	<0.001	1.14 (1.11–1.17)	<0.001	1.10 (1.08–1.12)	<0.001
**Multivariable (Model 1)**								
Systolic BP-CV (/1% increase)	1.09 (1.05–1.13)	<0.001	1.09 (1.05–1.14)	<0.001	1.11 (1.07–1.14)	<0.001	1.10 (1.08–1.12)	<0.001
Diastolic BP-CV (/1% increase)	1.01 (0.98–1.05)	0.528	1.03 (1.00–1.06)	0.089	1.07 (1.04–1.09)	<0.001	1.04 (1.02–1.06)	<0.001
**Multivariable (Model 2)**								
Systolic BP-CV (/1% increase)	1.07 (1.02–1.11)	0.002	1.09 (1.05–1.13)	<0.001	1.07 (1.04–1.11)	<0.001	1.10 (1.08–1.13)	<0.001
Diastolic BP-CV (/1% increase)	1.03 (0.99–1.06)	0.143	1.03 (1.00–1.07)	0.045	1.05 (1.02–1.08)	0.001	1.05 (1.03–1.07)	<0.001
**Multivariable (Model 3)**								
Systolic BP-CV (/1% increase)	1.09 (1.04–1.13)	<0.001	1.08 (1.04–1.13)	<0.001	1.09 (1.05–1.13)	<0.001	1.09 (1.06–1.11)	<0.001
Diastolic BP-CV (/1% increase)	1.00 (0.96–1.04)	0.933	1.02 (0.99–1.06)	0.241	1.04 (1.01–1.07)	0.012	1.03 (1.01–1.05)	0.013

* Thromboembolism, major hemorrhage, and all-cause death.
Model 1: adjusted for components of CHA_2DS_2-VASc score, warfarin and antiplatelet use, type of atrial fibrillation, BP measurement times.
Model 2: adjusted for variables of Model 1 plus BP at the time closest to the event or at the end of follow up.
Model 3: adjusted for variables of Model 1 plus creatinine clearance, body mass index, and hemoglobin level (N=5774).
BP, blood pressure; CV, coefficient of variation=standard deviation/mean; HR: hazard ratio; CI, confidence interval.

Table S10. Two-year event rates in each model of multivariable analysis.

	Model 1 (Overall)	Included in Model 3 (Including CrCl, BMI, and Hb)	Excluded from Model 3 (Missing CrCl, BMI, or Hb)	*P*-Value†
Number of patients	7226	5774	1452	
**Thromboembolism**	110 (1.5%)	95 (1.6%)	15 (1.0%)	0.089
**Major hemorrhage**	121 (1.7%)	98 (1.7%)	23 (1.6%)	0.764
**All-cause death**	168 (2.3%)	131 (2.3%)	37 (2.5%)	0.528
**Composite events***	399 (5.5%)	324 (5.6%)	75 (5.2%)	0.506

* Thromboembolism, major hemorrhage, and all-cause death.

† Comparison between included in and excluded from Model 3.

Data are number of patients (%).

CrCl, creatinine clearance; BMI, body mass index; Hb, hemoglobin.
